# A Novel Resource Allocation and Spectrum Defragmentation Mechanism for IoT-Based Big Data in Smart Cities

**DOI:** 10.3390/s19153443

**Published:** 2019-08-06

**Authors:** Yuhuai Peng, Jiaying Wang, Aiping Tan, Jingjing Wu

**Affiliations:** 1 School of Computer Science and Engineering, Northeastern University, Shenyang 110819, China; 2 Key Laboratory of Vibration and Control of Aero-Propulsion System of Ministry of Education, Northeastern University, Shenyang 110819, China

**Keywords:** Internet of Things (IoT), massive traffic, big data, spectrum defragmentation, optical backbone network

## Abstract

People’s demand for high-traffic applications and the need for Internet of Things (IoT) are enormous in smart cities. The amount of data generated by virtual reality, high-definition video, and other IoT applications is growing at an exponential rate that far exceeds our expectations, and the types of data are becoming more diverse. It has become critical to reliably accommodate IoT-based big data with reasonable resource allocation in optical backbone networks for smart cities. For the problem of reliable transmission and efficient resource allocation in optical backbone networks, a novel resource allocation and spectrum defragmentation mechanism for massive IoT traffic is presented in this paper. Firstly, a routing and spectrum allocation algorithm based on the distance-adaptive sharing protection mechanism (DASP) is proposed, to obtain sufficient protection and reduce the spectrum consumption. The DASP algorithm advocates applying different strategies to the resource allocation of the working and protection paths. Then, the protection path spectrum defragmentation algorithm based on OpenFlow is designed to improve spectrum utilization while providing shared protection for traffic demands. The lowest starting slot-index first (LSSF) algorithm is employed to remove and reconstruct the optical paths. Numerical results indicate that the proposal can effectively alleviate spectrum fragmentation and reduce the bandwidth-blocking probability by 44.68% compared with the traditional scheme.

## 1. Introduction

As the foundation of smart cities, Internet of Things (IoT) has become the cornerstone for deepening the construction of the “urban brain”, in which numerous intelligent sensors, devices, and vehicles are connected to realize continuous coverage of urban areas and high-capacity coverage of key scenes [1,2]. In smart cities, people’s demand for mobile Internet high-traffic applications and the need for IoT are enormous. This big-data-driven IoT would be the main driving force for the fifth-generation (5G) infrastructure deployment in smart cities, and also the industry believes that 5G can provide comprehensive support for ubiquitous IoT applications [3,4]. It is estimated that by 2021, there will be 28 billion mobile devices connected, of which 16 billion will be IoT-based sensors [5]. The amount of data generated by numerous sensors is growing at an exponential rate that far exceeds our expectations, and the types of data are becoming more diverse. The backbone infrastructure must meet the high requirements of high-speed, real-time, massive, stable, and other communication networks in mobile services such as virtual reality, high-definition video, and other IoT applications in smart cities [6,7].

The frequently-changing big data produced by numerous sensors has posed a great challenge to resource allocation, network optimization, multimedia data sensing and processing, etc. This IoT-based big data traffic requires optical backbone networks to carry reliably and efficiently [8,9]. However, spectrum resources are frequently reconstructed and released, resulting in severe spectrum fragmentation. Those unaligned, isolated, small-sized frequency slots (FS) blocks are difficult to use by the path, which reduces service admissibility and thus affects the quality of network operation [10]. It has become critical to reliably accommodate the IoT-based big data with a reasonable resource allocation method. Moreover, it would be better to make a trade-off between additional spectrum consumption, due to establishing protection paths, and increase of congestion probability, caused by excessive spectrum utilization in optical backbone networks for smart cities.

Researches on existing resource allocation approaches in optical backbone networks are mostly concerned about the working path to defragment, with little regard for the protection path to be defragmented. Focusing on the efficient spectrum allocation for reliable transmission, we propose a novel resource allocation and spectrum defragmentation (DF) mechanism for IoT-based big data. The main contributions of this paper can be listed as follows:

(1) The distance-adaptive modulation (DAM) technology is employed to assign different modulation formats and different numbers of FS to the working and protection paths in optical backbone networks. In particular, a routing and spectrum allocation algorithm based on the distance-adaptive sharing protection mechanism (DASP) is proposed, which can reduce the spectrum consumption of the protection path while providing protection for services.
(2) For network congestion caused by spectrum fragmentation under massive IoT-based big data, the online spectrum defragmentation algorithm based on OpenFlow is designed to reconfigure the protection paths. Furthermore, the spectrum resource utilization with reliability constraint is also optimized.

The rest of the paper is organized as follows: Section 2 reviews the research progress of the routing and spectrum allocation (RSA), and DF methods. Design of the network framework and function model based on software-defined networking (SDN) are then presented in Section 3. Section 4 describes the DASP algorithm for meeting the big data needs of massive IoT traffic. The strategy for spectrum defragmentation of the protection paths is described in Section 5. In Section 6, we make a comparison of our proposed scheme with the traditional schemes and evaluate their bandwidth-blocking probability (BBP) performance. The last section is the conclusion of this paper.

## 2. Related Works

Recently, many different resource allocation and spectrum defragmentation approaches have been proposed to achieve efficient resource utilization in optical backbone networks. In this section, we shall review the existing work of the RSA and DF schemes.

### 2.1. Routing and Spectrum Allocation

In recent years, to find a solution that can improve network transmission capacity and spectrum utilization in optical backbone networks, the elastic optical networks based on orthogonal frequency division multiplexing (OFDM) has emerged [11]. The entity of resource allocation changes from wavelength to spectrum, and the allocation strategy of resource management and regulation problems becomes complicated [12,13]. For the RSA issue in optical backbone networks, it can be extended to multiple issues such as routing, modulation level, and spectrum allocation. The authors in [14] considered distance-adaptive dynamic routing and spectrum assignment (RSA) for elastic optical networks with shared backup path protection (SBPP). Their efficient heuristic algorithms based on spectrum window planes were proposed for implementing distance and modulation format-adaptive RSA, so as to maximize spare capacity sharing among multiple protection paths. The authors of [15] proposed two dynamic RSA algorithms based on K shortest path (KSP); the algorithm needs to determine the candidate path in advance. In [16], the improved shortest path (MSP) algorithm and the spectrum constrained path vector search (SVP) algorithm were addressed, which can further improve network performance. To some degree, it will be at the expense of higher complexity. The authors of [17] proposed a dynamic RSA algorithm based on multi-path routing, which allows a demand to be divided into multiple requirements and transmitted on multiple paths. In [18,19], the authors investigated a novel method to evaluate clustering algorithms for hierarchical optical networks, they also presented a novel framework and the application mechanism with cooperation of control and management. The authors in [20] designed a simulator that can run and compare the performance of various RSA algorithms for studying RSA problems in optical backbone networks.

### 2.2. Spectrum Defragmentation

For the spectrum defragmentation (DF) issue in optical backbone networks, several defragmentation approaches have been presented. A.N. Patel et al. proposed two algorithms, namely “greedy-defragmentation” and “shortest path-defragmentation algorithm” [21]. In order to describe the basic idea of traffic splitting strategy in one-way resource allocation, the authors in [22] proposed a mathematical model with heuristic algorithm to accelerate resource allocation. In [23], the first-fit (FF) allocation strategy is used to assign light paths to partitions defined according to links used by particular connections. With most of the reactive approaches leading to traffic disruption and the proactive ones often providing no reactive measure to counter additional fragmentation induced by expired connection, hitless defragmentation was considered in [24]. The authors in [25] designed a defragmentation-based load balancing technique, which efficiently undertakes routing and spectrum assignment (RSA) and minimizes the fragmentation problem in an elastic optical network. In [26], the influence of different defragmentation parameters on network performance were analyzed.

### 2.3. Survivability in Optical Backbone Networks

For the dynamic optical communication requests, a dynamic distance-based adaptive shared-protection path strategy was proposed in [27]. The heuristic algorithm can effectively reduce the BBP of network services. In [28], pre-configured polyhedron-based protection was proposed to aid against multi-link failures in optical mesh networks. Shao et al. in [29] proposed a shared path-protection (SPP) based on the routing strategy of K shortest path and the spectrum allocation strategy of first-fit (FF). The task scheduling and resource allocation strategy is formulated in [30] as a joint optimization problem to maximize users’ quality of experience (QoE). The authors in [31] proposed a defragmentation scheme using path switching in an optical network based on 1 + 1 path protection. Content-aware filtering for security services in centric networks was proposed in [32,33], it can realize the virtualization of network functions and better adapt to the needs of the future Internet. The above research results indicate that shared protection can achieve high free-capacity-sharing efficiency and improve spectrum utilization.

### 2.4. SDN based Approaches

Software-defined networking (SDN) decouples the control plane and data plane of the network, making the network programmable, adaptive, and application aware. The authors in [34] presented a new approach for comprehensive monitoring of a software-defined 5G mobile network by using an IoT (Internet of Things)-based framework. The proposed framework provides mobile network operators with an easier implementation of monitoring systems. SDN support centralized the control of network control and management, giving service providers greater freedom to dynamically define network devices [35]. The authors in [36] proposed a new interoperability backup model to improve the survivability of the control plane in SDN. However, in order to consider survivability, the allocation of spectrum resources needs further research.

## 3. Overall Network Architecture and System Model

In optical backbone networks, to efficiently and intelligently manage network spectrum resources and improve spectrum utilization, the control plane can be used for defragmentation. One of the candidate control plane schemes is the SDN, which manages the global information of the network, including the network topology and spectrum usage on each link. As one of the most promising implementations of SDN, OpenFlow (OF) is a key protocol proposed by researchers at Stanford University for SDN architecture, which utilizes stream-based transformation and uses a centralized controller to facilitate software-defined routing, transformation, and network management.

### 3.1. Design of SDN-Based Network Architecture

**Figure 1:**
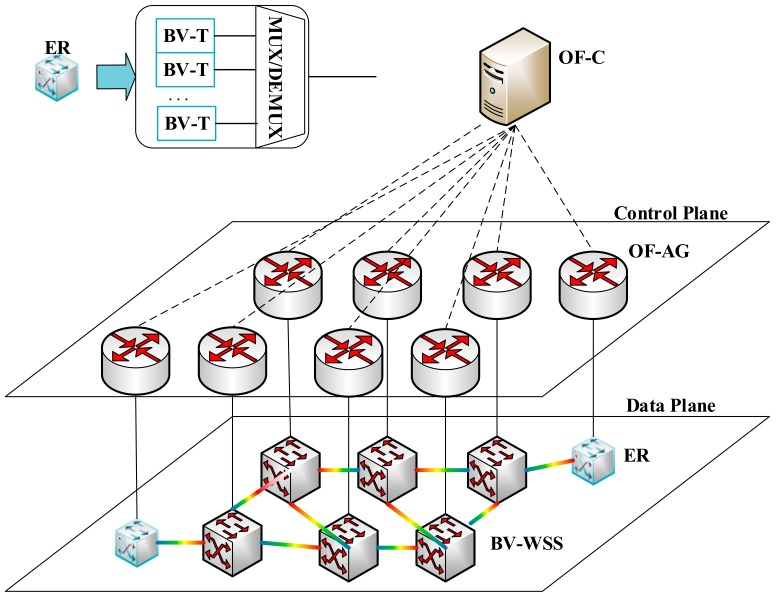
SDN-based network architecture.

The SDN-based network architecture shown in Figure 1 consists of the data plane and control plane. The data plane includes an edge router (ER) and a bandwidth-variable wavelength selective switch (BV-WSS). When serving the traffic demands, the bandwidth-variable transponder (BV-T) in the ER allocates appropriate spectrum resources for client traffic, and the BV-WSS performs data forwarding according to the corresponding frequency band. Above the data plane, the OpenFlow-based control plane is responsible for centralized and efficient resource management of the entire data plane. The control plane consists of a centralized OpenFlow controller (OF-C) and several OpenFlow agents (OF-AG), which communicate with each other through the extended OpenFlow protocol. Each data plane device has an OpenFlow agent connected to it locally to control the dynamic optical path establishment and deletion of the data plane.

### 3.2. Description of System Model

**Figure 2:**
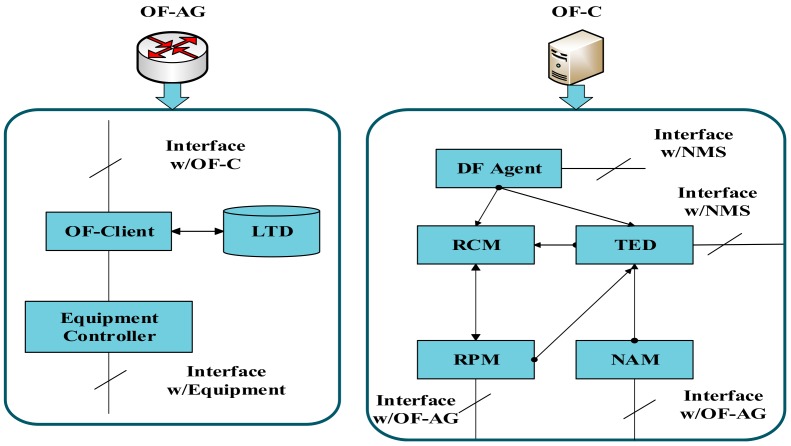
SDN-based system function module, OF-AG: Openflow Agent, OF-C: Openflow Controller, OF-Client: Openflow Client, LTD: Local Traffic Database, DF Agent: Defragmentation Agent, NMS: Network Management System, RCM: Resource Computation Module, TED: Traffic Engineering Database, RPM: Resource Provisioning Module, NAM: Network Abstraction Module.

Figure 2 shows the functional design of the OF-AG and the OF-C. The OF-AG is a bridge between the control plane and the data plane. The data plane device needs to be configured according to the flow table from the OF-C. The OpenFlow client (OF-Client) uses the extended OF to communicate with the OF-C. The local traffic database (LTD) stores the flow table, and the device controller implements the flow table of the data plane device. As the “brain” of the SDN, the OF-C needs to intelligently determine the optical path configuration strategy according to the network status. In the OF-C function module, the resource provision module (RPM) communicates with the OF-AG and the resource calculation module (RCM) to process the OF information. The resource computation module (RCM) receives computing tasks from the RPM or the DF-AG and calculates the corresponding RSA solution. When the OF-C receives the request from the OF-AG, the RPM receives the request message, parses it and hands it to the RCM for processing. In addition, the RCM also receives requests from the defragmentation agent (DF Agent) for spectrum defragmentation operations. The DF-AG invokes the DF operation to select the optical path in the current service for reconfiguration, instructing the RCM to calculate a new RSA solution for it. Both DF-AG and TED are externally connected to an external network management system (NMS). DF-AG can accept NMS defragmentation requests, and TED can accept NMS content reading and monitoring. The network abstraction module (NAM) is responsible for communicating with each OF-AG, collecting topology information in the network, and abstracting all data plane device information into the TED.

## 4. Design of DASP Mechanism

In order to improve spectrum utilization, this paper proposes a routing and spectrum allocation algorithm based on the distance-adaptive sharing protection mechanism (DASP) in optical backbone networks for smart cities. For each traffic demand, Dijkstra’s shortest path algorithm is used to search for working and protection routes, the first-fit (FF) strategy and the least shared cost (LSC) strategy are applied to allocate spectrum resources for the working and protection paths, respectively. Considering the case where the spectrum of the optical transceiver is adjustable, the frequency slots on the working path and the protection path can use different index values.

A summary of the notations used in this paper is presented in Table 1.

### 4.1. Distance-Adaptive Modulation (DAM)

Network survivability is a critical networking problem in optical backbone networks. Establishing a protection path for services can effectively improve network survival performance, but it is not conducive to efficient use of spectrum resources. In order to alleviate this problem, in the process of establishing work and protection paths, the DAM technology is used to adaptively select the modulation format suitable for the transmission distance for the working and protection paths [37]. Specifically, when a low-order modulation format (such as binary phase shift keying, that is, BPSK) is used for transmission, the bandwidth of the signal is increased, and the anti-interference capability is also enhanced. As shown in Table 2, for every 1-bit modulation format added in the backbone network, the amount of traffic information that can be carried by the unit frequency slot will be doubled, and the transmission distance will be reduced to half. (The subcarrier load capacity in the table indicates the FS capacity under the corresponding modulation format, Quadrature Phase Shift Keying (QPSK), Quadrature Amplitude Modulation (QAM)).

Due to transmission loss and physical distance limitations, working and protecting paths may use different modulation formats. The modulation format is determined by the actual length of each connection path. Since low modulation levels result in more spectrum consumption, we try to choose a high modulation level. In order to describe the DASP algorithm in detail, the decision of the modulation level in the RSA is first illustrated. We can use an eight-node graph to represent the network topology. Here we uses G(V,E) to represent an elastic optical network, where V represents a collection of physical nodes and E represents a collection of fiber links. A service connection request is represented as $C_{i}(S_{i},D_{i},R_{i})$, $S_{i}$ is the source node, $D_{i}$ is the destination node, $R_{i}$ is the traffic demand transmission rate, and the unit is Gbit/s. Assuming that the bandwidth per FS is 12.5 GHz, the capacity of one traffic demand is an integer multiple of the FS size. To achieve the required bandwidth R for the traffic demand, the required number of FSs can be calculated according to formula F=R/ME for a certain modulation format, where ME represents the carrying capacity per FS of the modulation format, corresponding to BPSK, QPSK, 8-QAM, and 16-QAM, which are 12.5, 25, 37.5, and 50 Gbit/s, respectively.

**Figure 3:**
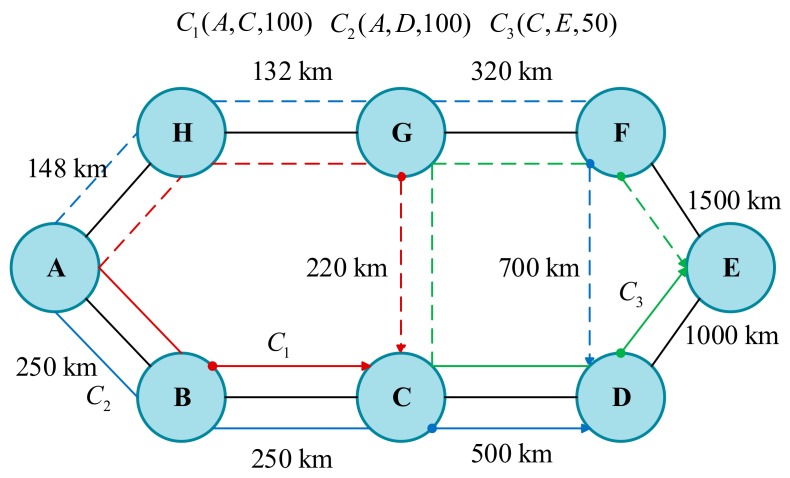
An eight-node network.

**Figure 4:**
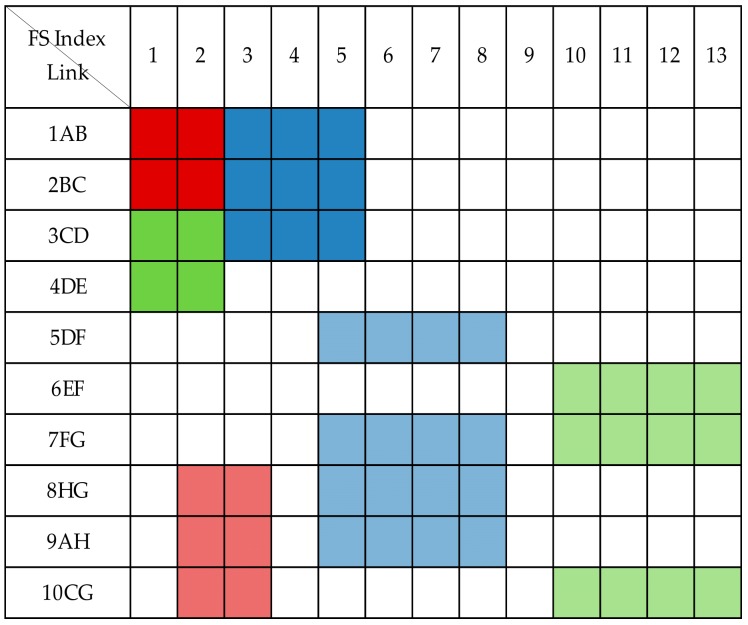
Spectrum usage status without spectrum sharing.

We use an example to illustrate the application of DAM technology in spectrum resource allocation, which considers an eight-node network as shown in Figure 3, with the current state of network spectrum resources as shown in Figure 4. When the first request $C_{1}(A,C,100)$ arrives, the working path assigned to this request is the shortest path A−B−C calculated by the Dijkstra’s shortest path algorithm, with a physical distance of 500 km. Since the working optical path distance of $C_{1}$ is short, the more efficient modulation format 16-QAM can be selected, and the spectrum resources of [100/50]=2 frequency slots are needed. The distance of its protection path A−H−G−C is 500 km, and we select 16-QAM and allocate two frequency slots for the protection path. Similarly, when the second request $C_{2}(A,D,100)$ arrives, its working path is A−B−C−D, the distance is 1000 km, the appropriate modulation format is 8-QAM, and the required number of FS is [100/37.5]=3. Its protection path is A−H−G−F−D, the distance is 1300 km, the modulation format is QPSK, and the required number of FS is 4. When the third request $C_{3}(C,E,50)$ arrives, the working path is A−H−G−F−D, the distance is 1500 km, we need to select the modulation format QPSK for it, and the number of FS is [50/25]=2. The length of its protection path C−G−F−E is 2040 km, the modulation format is BPSK, and the required number of FS is 4.

### 4.2. Shared Protection Principle

Protection technologies in optical backbone networks are mainly divided into dedicated protection and shared protection technologies. The DASP algorithm considers shared backup path protection—a path-oriented protection technique. We need to preset the protection path and protection capacity in advance. When a failure occurs, the service will be switched to the corresponding protection path for transmission [38,39].

**Figure 5:**
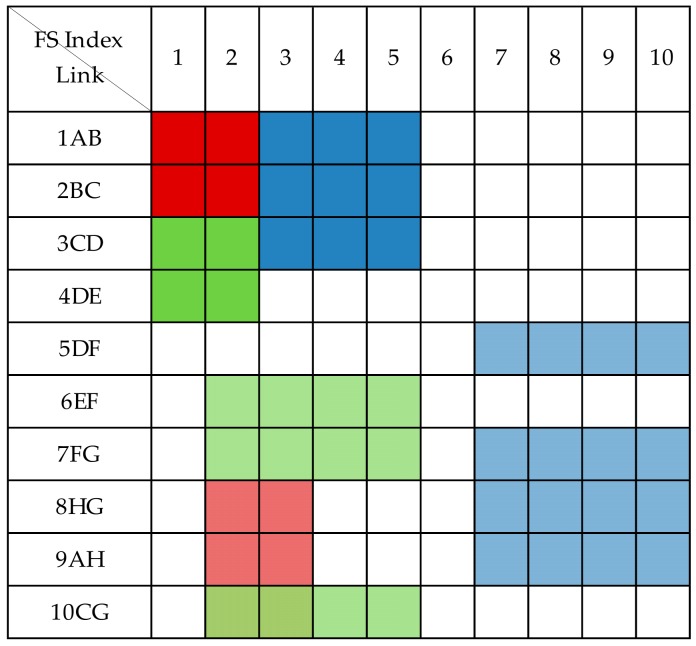
Spectrum usage status when spectrum sharing.

The shared protection allows spare capacity sharing among multiple protection paths so long as their corresponding working paths cannot share capacity with other services. In the 1 + 1 dedicated protection technology, the protection capacity of each optical path is dedicated to protecting its corresponding working optical path. Compared with the 1 + 1 dedicated protection technology, shared backup path protection can more effectively utilize protection resources and improve spectrum utilization. As shown in Figure 3, the CG segments of $C_{1}$ and $C_{3}$ can share spectrum resources because their protection paths do not intersect. The spectrum resource usage status under shared protection is shown in Figure 5. Compared with Figure 4, the maximum index occupying FS is changed from 13 to 10, and two FSs are saved. This shows that shared protection achieves high free-capacity-sharing efficiency and improves spectrum utilization.

### 4.3. Routing and Spectrum Allocation Strategy

In the SDN framework, OF-C controls the entire networks. When a traffic demand arrives, an RSA scheme is calculated for the request and an optical path is established. In this paper, the RCM adaptively selects the modulation level and calculates the RSA scheme by using the DASP algorithm when processing the RSA task. In the DASP algorithm, the distance-adaptive modulation (DAM) technology is employed to adaptively select the appropriate modulation format for the services. For each traffic demand, the DASP algorithm advocates applying different strategies to the resource allocation of the working and protection paths. Specifically, we use the first-fit (FF) strategy for the working path and the least shared cost (LSC) strategy for the protection path. The DASP algorithm makes the services tend to use sharable spectrum resources, which can reduce the waste of spectrum resources caused by establishing the protection paths.

In the routing step, the route with the lowest cost is considered the one with the fewest hops. The available frequency slot of the protection path may be an unoccupied FS, or an FS that is used by the protection path of other services but whose working links do not intersect. The DASP algorithm advocates applying different algorithms to the resource allocation of the working and protection paths in spectrum allocation. Specifically, we use the first-fit (FF) strategy for the working path and the least shared cost (LSC) strategy for the protection path, with the aim of reducing fragmentation and improving spectrum utilization. Under the FF strategy, we will select the first available path and continuous frequency slot blocks. In order to obtain the best network performance, this section proposes a novel LSC strategy for allocating spectrum resources for the protection path. The LSC strategy compares all candidate continuous frequency slots and selects the lowest sharing cost case for establishing the optical path. 

The LSC strategy considers the sharing state of each FS to set the cost value, so that the more times of sharing, the smaller the cost value. The cost of the $j^{th}$ frequency slot $f_{j}$ in a continuous FS block is defined in Equation (1):

(1) $$f_{j}=\left\{\begin{array}{l} 1,\;if\;the\;FS\;is\;not\;occupied\\ 1/m_{j}+1,\;if\;the\;FS\;can\;be\;shared \end{array}\right..$$

Then, to find a continuous FS block established for the traffic demand $C_{i}$ with the lowest cost, the cost is calculated in Equation (2):

(2) $$W_{i}=\sum_{k\in P}\sum_{j=1}^{F}f_{j},$$

where $m_{j}$ is the number of protection paths sharing the $i^{th}$ FS in a contiguous FS block, F is the number of FSs in a continuous FS block, k is the $k^{th}$ link in set E, and P is the set of links through which the protection optical path established for the traffic demand $C_{i}$ passes. In order to achieve better network performance, the LSC strategy will make the upcoming traffic demands more inclined to use FS blocks with smaller cost values.

Based on the above introduction, the pseudo-code for DASP algorithm details are described as Algorithm 1.

**Algorithm 1** Pseudo-Code for DASP Algorithm1: DASP(C, G(V,E)){//Start of function DASP 2: **if**
$C_{i}(S_{i},D_{i},R_{i})$ is for establishing a new connection **then** 3: **for**
$C_{i}(S_{i},D_{i},R_{i})$ in C
**do** 4:   Run Dijkstra on G to calculate $WP(S_{i},D_{i},R_{i})$; 5:   **if** it is successful **then** 6:    **for** (j=0; $j<L_{WP}$; j++) **do** 7:     Determine the modulation format for $WP(S_{i},D_{i},R_{i})$; 8:     Calculate $F_{i}$;//according to F=R/ME; 9:     Run FF to allocate spectrum resources for $WP(S_{i},D_{i},R_{i})$; 10:      **if** it is successfully allocated **then** 11:       Update the spectrum resource usage status; 12:       Remove $WP(S_{i},D_{i},R_{i})$ from G; 13:       break;//exit the RSA of $WP(S_{i},D_{i},R_{i})$ 14:      **else** remove $WP(S_{i},D_{i},R_{i})$ from $L_{BP}$; 15:      **end if** 16:      **if**
$L_{WP}=\phi$
**then** 17:       Block $C_{i}(S_{i},D_{i},R_{i})$ in $C_{B}$ and exit the entire program; 18:      **end if** 19:    **end for** 20:   Run Dijkstra on G to calculate $PP(S_{i},D_{i},R_{i})$; 21:     **if** it is successful **then** 22:      **for** (k=0; $k<L_{PP}$; k++) **do** 23:      Determine the modulation format for $PP(S_{i},D_{i},R_{i})$; 24:      Calculate $F_{i}$;//according to F=R/ME; 25:      Run LSC to allocate spectrum for $PP(S_{i},D_{i},R_{i})$;//according to Equation (2) 26:       **if** it is successfully allocated **then** 27:       Update the spectrum resource usage status; 28:       **break**;//the RSA of $WP(S_{i},D_{i},R_{i})$ and $PP(S_{i},D_{i},R_{i})$ is completed 29:       **else** remove $PP(S_{i},D_{i},R_{i})$ from $L_{PP}$; 30:       **end if** 31:        **if**
$L_{PP}=\phi$
**then** 32:         Release the spectrum resources occupied by $WP(S_{i},D_{i},R_{i})$, block $C_{i}(S_{i},D_{i},R_{i})$ in $C_{B}$ and exit the entire program; 33:        **end if** 34:      **end for** 35:     **end if** 36:   **end if** 37: **end for** 38: **else**
$C_{i}(S_{i},D_{i},R_{i})$ is for releasing an old connection **then** Remove $C_{i}(S_{i},D_{i},R_{i})$ from the network; 39:   Release the remaining spectrum resources; 40: **end if** 41:**}** The algorithm ends.

## 5. Protection Path Spectrum Defragmentation Scheme

In order to alleviate spectrum fragmentation, we propose an online protection path spectrum defragmentation algorithm LSSF based on OpenFlow. The LSSF algorithm first attempts to remove and reconstruct the protection paths with a lower start index value of the FSs. When performing spectrum rearrangement, the algorithm selects the lowest starting slot-index available spectrum resources that satisfy the traffic demand. The LSSF algorithm can effectively alleviate the problem of spectrum fragmentation and further improve spectrum utilization.

Under the dynamic traffic demands in optical backbone networks, the continuous establishment and removal of optical paths and the continuous evolution of the spectrum can cause severe spectrum fragmentation. In order to alleviate spectrum fragmentation, defragmentation must be done frequently. The purpose of spectrum fragmentation is to concentrate the spectrum resources occupied by the services, and keep the unoccupied spectrum resources continuous to reduce the situation whereby requests are blocked due to fragmentation. In this section, based on the DASP algorithm, the OpenFlow is used to manage the protection optical paths, and the lowest starting slot-index first (LSSF) algorithm is used to remove and reconstruct the optical paths. Specifically, the OF-C actively collects and analyzes the spectrum usage on each link. When defragmentation is required, the OF-C evokes a defragmentation operation to notify all the corresponding OF-AGs to change the working state of the data plane equipment. To reconfigure the light path, the LSSF algorithm is described as Algorithm 2.

**Algorithm 2** Pseudo-Code for LSSF Algorithm1: LSSF($C,S_{i},S_{i}[M],L[K]$){//Start of function LSSF 2://$S_{i}$ is the original starting slot-index of the protection path spectrum resource block, i represents $i^{th}$ traffic demand in C. 3://$S_{i}[M]$ is an array of the starting slot-index of the available spectrum resource block, the number of the available spectrum resource block is M−1. 4://L[K] is the list of protection paths, the length of it is K−1. 5: **for**(int k=0; k<K; k++)//start of K-loop 6:  **for**(int m=0; m<M; m++)**do**//start of M-loop 7:    **if**($S_{i}[M]<S_{i}$)**then** 8:     $S_{i}=S_{i}[M]$; 9:    **end if** 10:  **end for** 11: return $S_{i}$; 12: **end for** 13: return C; 14: return $C_{B}$; 15:**}** The algorithm ends.

Compared with the FF algorithm, the LSSF algorithm has great advantages, this is because the LSSF algorithm can utilize any frequency slot block wider than the required spectrum, and the utilization rate is greatly increased. The LSSF algorithm first attempts to remove and reconstruct the protection optical paths service with a lower start index value of the FSs. The currently established protection optical paths are arranged in ascending order according to the FS starting index, and each protection optical path service is sequentially removed and reconstructed. When reconstructing the optical path, we make the new spectral position of an optical path more forward. When an optical path cannot be reassigned to a lower allocation index, the defragmentation operation is abandoned. The LSSF algorithm maintains an index list of available frequency slots, scanning one by one from the lowest indexed spectrum resources, and selecting the lowest starting slot-index available spectrum resources that satisfy the traffic demand. By selecting the spectrum in this way, the current connection request is allocated to the frequency slot resources with a small number of index values, which allows the optical paths allocated on the higher spectral index to be reallocated to the unoccupied spectrum left by them.

**Figure 6:**
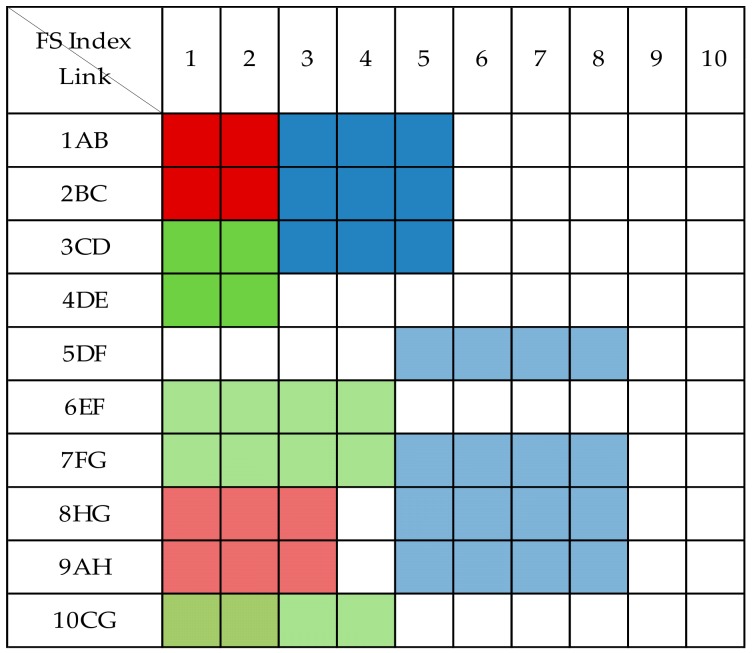
Spectrum resource usage status after defragmentation.

Figure 6 shows the spectrum resource state after the defragmentation of the three services $C_{1},C_{2},C_{3}$. According to the LSSF scheme, we should first assign $C_{1}$ and $C_{3}$ with lower frequency slot start index values, and then assign $C_{2}$.

## 6. Numerical Results and Analysis

### 6.1. Simulation Parameter Settings

The BBP performance of the proposed RSA and defragmentation algorithms is evaluated via simulations. We usethe high-performance server of the laboratory to build the SDN control plane experimental platform, including a centralized controller OF-C and multiple OF-AGs. We consider the NSFNET network of 14 nodes and 21 links and the USNET network topology of 24 nodes and 43 links to interconnect them. Assume that there is a dynamic optical path service model with a total bandwidth of 5000 Hz available in each fiber, each FS has a bandwidth of 12.5 GHz and there are 400 FSs per fiber link. The service arrival rate of each node obeys the Poisson distribution with the parameter λ. The hold time of each service follows the negative exponential distribution with the parameter 1/μ. The traffic load is the product of the service arrival rate and the average service duration λ/μ. The bandwidth requirements of each rate service are 25, 50, 50, and 75 GHz, respectively. The number of FSs required for each request is determined adaptively based on distance. In order to verify the advantages and disadvantages of the proposed algorithm, the bandwidth-blocking rate is taken as the main evaluation index of network performance. A total of $10^{5}$ light-path arrival events are simulated to calculate BBP. BBP is defined as the ratio of the amount of blocked service bandwidth to the total amount of arriving services bandwidth, we calculate it in Equation (3) as follows:

(3) $$BBP=\frac{\sum_{i\in C_{B}}R_{i}}{\sum_{i\in C}R_{i}},$$

where $R_{i}$ represents the bandwidth requirement of the $i^{th}$ service, C represents the set of all arriving traffic demands, and $C_{B}$ represents the set of all blocked traffic demands. 

### 6.2. Simulation Results and Analysis

For performance comparisons, we evaluate the results for different situations. “1 + 1-FF” means that the FF strategy is used to allocate the dedicated protection optical path spectrum without considering the distance constraint, and “DASP-FF” is an algorithm for assigning the protection optical path spectrum by using the FF strategy in consideration of the distance constraint. “DASP-LSC” means an algorithm that uses the LSC policy to allocate the spectrum of the protection optical paths in consideration of the distance constraint.

**Figure 7:**
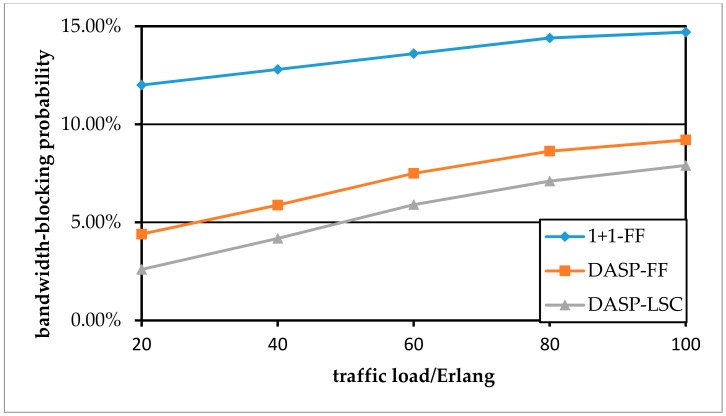
Comparison of BBP for various RSA scenarios in the NSFNET network.

**Figure 8:**
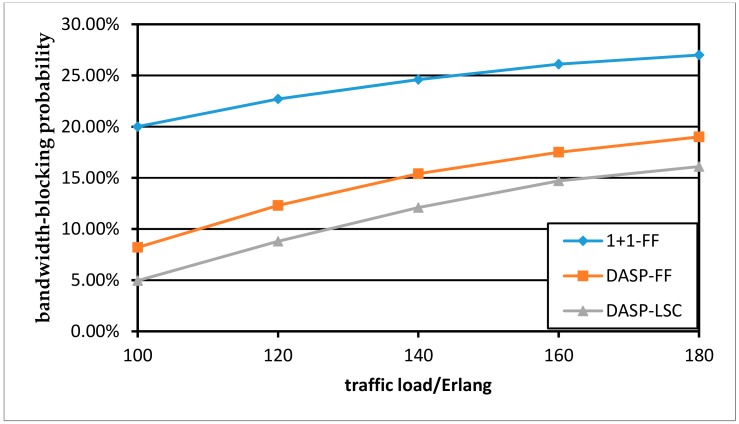
Comparison of BBP for various RSA scenarios in the USNET network.

Figure 7 and Figure 8 show how the BBP performance changes with the increase of traffic load per node pair. As more traffic demands arrive, there are not enough free spectrum resources in the network to provide for subsequent arriving services. The shared protection RSA algorithm considering the distance constraint can outperform the case “1 + 1-FF”. In addition, compared to the FF strategy, the LSC strategy improves the performance of BBP better. This is because the more FSs that are shared by the protection optical path, the smaller the cost value is, and the future protection optical paths tend to use these low-cost FSs under the LSC strategy. However, the strategy comes at the cost of more computing time.

**Figure 9:**
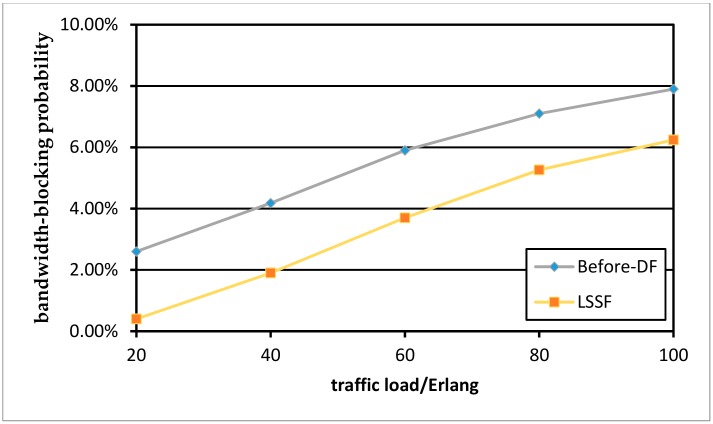
BBP with LSSF algorithm in the NSFENT network.

**Figure 10:**
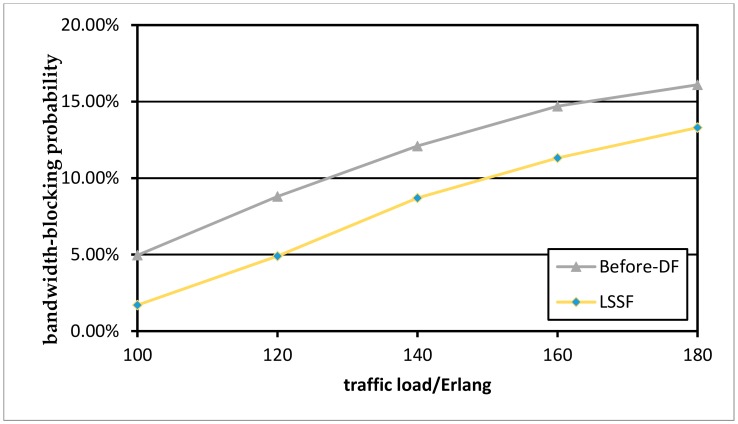
BBP with LSSF algorithm in the USNET network.

Next, we compare the situation before and after defragmentation of the protection paths. Figure 9 shows the comparison of BBP without defragmentation and defragmentation with the LSSF algorithm in the NSFENT network. Figure 10 is an experimental result of the USNET network topology. Although the USNET has a larger topology size, more network nodes, and a larger number of links, the simulation results are similar to those of the NSFENT network. As can be seen in the two figures, the LSSF scheme has an obvious reduction in BBP, it can significantly outperform the “Before-DF” case. The defragmentation of the protection paths will not affect the established optical transmission of the working paths while improving the spectrum utilization.

## 7. Conclusions

In smart cities, all the intelligent things including billions of sensors, devices, and vehicles are generating massive data calculated in ZettaBytes, and the types of data are becoming more diverse. The big data generated by virtual reality, high-definition video, and other IoT applications has posed a great challenge to efficient resource allocation in optical backbone networks for smart cities. For the problem of reliable transmission and efficient resource allocation in optical backbone networks, this paper focuses on the study of resource allocation and the spectrum defragmentation mechanism to relieve network congestion and improve spectrum utilization. Firstly, a routing and spectrum allocation algorithm based on distance-adaptive sharing protection is proposed. To improve spectrum utilization while providing protection for services, an online defragmentation algorithm for protecting paths is then designed. The simulation results indicate that the proposal has a 44.68% reduction in bandwidth-blocking probability compared to the traditional scheme.

## Figures and Tables

**Table 1:** Summary of notations.

Notation	Description
G(V,E)	The network topology
V	Set of physical nodes
E	Set of fiber links
C	Set of traffic requests
$C_{B}$	Set of blocked traffic requests
$C_{i}(S_{i},D_{i},R_{i})$	The $i^{th}$ traffic request
$WP(S_{i},D_{i},R_{i})$	The working path of $C_{i}(S_{i},D_{i},R_{i})$
$PP(S_{i},D_{i},R_{i})$	The protection path of $C_{i}(S_{i},D_{i},R_{i})$
$S_{i}$	The source node of $C_{i}$
$D_{i}$	The destination node of $C_{i}$
$R_{i}$	The traffic request bandwidth of $C_{i}$
F	Number of FSs required
ME	The carrying capacity per FS of the modulation format
$f_{j}$	The cost of the $j^{th}$ frequency slot
$m_{j}$	Number of protection paths sharing the $i^{th}$ FS in a contiguous FSs block
k	The $k^{th}$ link in set E
P	Set of links through which the protection path established for $C_{i}$ passes
$L_{WP}$	The list of the available $WP(S_{i},D_{i},R_{i})$
$L_{PP}$	The list of the available $PP(S_{i},D_{i},R_{i})$

**Table 2:** Subcarrier capacity and longest transmission distance in different modulation formats.

Modulation Format	Subcarrier Load Capacity/Gb	Longest Transmission Distance/km
BPSK	12.5	4000
QPSK	25	2000
8-QAM	37.5	1000
16-QAM	50	500
32-QAM	62.5	250
64-QAM	75	125
